# Phenethyl Isothiocyanate Inhibits In Vivo Growth of Xenograft Tumors of Human Glioblastoma Cells

**DOI:** 10.3390/molecules23092305

**Published:** 2018-09-10

**Authors:** Yu-Cheng Chou, Meng-Ya Chang, Hsu-Tung Lee, Chiung-Chyi Shen, Tomor Harnod, Yea-Jiuan Liang, Rick Sai-Chuen Wu, Kuang-Chi Lai, Fei-Ting Hsu, Jing-Gung Chung

**Affiliations:** 1 Department of Neurosurgery, Neurological Institute, Taichung Veterans General Hospital, Taichung 40754, Taiwan; chouycns@yahoo.com.tw (Y.-C.C.); leesd2001@gmail.com (H.-T.L.); shengeorge@yahoo.com (C.-C.S.); shelby63liang@gmail.com (Y.-J.L.); 2 Department of Neurological Surgery, Tri-Service General Hospital, National Defense Medical Center, Taipei 114, Taiwan; 3 Rong Hsing Research Center for Translational Medicine, National Chung Hsing University, Taichung 402, Taiwan; 4 Institute of Medical Sciences, Tzu Chi University, Hualien 970, Taiwan; mchang@mail.tcu.edu.tw; 5 Graduate Institute of Medical Sciences, National Defense Medical Center, Taipei 11490, Taiwan; 6 Department of Physical Therapy, Hung Kuang University, Taichung 43302, Taiwan; 7 Department of Neurosurgery, Hualien Tzu Chi General Hospital, Buddhist Tzu Chi Medical Foundation, Hualien 970, Taiwan; tomorha@yahoo.com.tw; 8 College of Medicine, Tzu Chi University 970, Hualien, Taiwan; 9 Department of Anesthesiology, China Medical University Hospital, Taichung 404, Taiwan; rickwu@mail.cmuh.org.tw; 10 Department of Anesthesiology, China Medical University, Taichung 40402, Taiwan; 11 Department of Medical Laboratory Science and Biotechnology, College of Medicine and Life Science, Chung Hwa University of Medical Technology, Tainan 71703, Taiwan; kuangchi_lai@hotmail.com; 12 Departments of Biological Science and Technology, China Medical University, Taichung 40402, Taiwan; 13 Department of Biotechnology, Asia University, Taichung 413, Taiwan

**Keywords:** phenethyl isothiocyanate, apoptosis, xenograft, caspase, glioblastoma

## Abstract

Phenethyl isothiocyanate (PEITC) from cruciferous vegetables can inhibit the growth of various human cancer cells. In previous studies, we determined that PEITC inhibited the in vitro growth of human glioblastoma GBM 8401 cells by inducing apoptosis, inhibiting migration and invasion, and altering gene expression. Nevertheless, there are no further in vivo reports disclosing whether PEITC can suppress the growth of glioblastoma. Therefore, in this study we investigate the anti-tumor effects of PEITC in a xenograft model of glioblastoma in nude mice. Thirty nude mice were inoculated subcutaneously with GBM 8401 cells. Mice with one palpable tumor were divided randomly into three groups: control, PEITC-10, and PEITC-20 groups treated with 0.1% dimethyl sulfoxide (DMSO), and 10 and 20 μmole PEITC/100 μL PBS daily by oral gavage, respectively. PEITC significantly decreased tumor weights and volumes of GBM 8401 cells in mice, but did not affect the total body weights of mice. PEITC diminished the levels of anti-apoptotic proteins MCL-1 (myeloid cell leukemia 1) and XIAP (X-linked inhibitor of apoptosis protein) in GBM 8401 cells. PEITC enhanced the levels of caspase-3 and Bax in GBM 8401 cells. The growth of glioblastoma can be suppressed by the biological properties of PEITC in vivo. These effects might support further investigations into the potential use of PEITC as an anticancer drug for glioblastoma.

## 1. Introduction

Aside from surgery, chemotherapy with the alkylating agent temozolomide and radiotherapy are first-line therapies for glioblastoma, but their survival benefits are short-lived and the tumors may develop resistance to therapies [1]. This most common and aggressive primary brain malignancy cannot be well-controlled by the current multimodality treatments. Phenethyl isothiocyanate (PEITC), a component in cruciferous vegetables, has chemopreventive activity for various tumors [2], and has also been applied in small human clinical trials against different diseases from cancer to autism [3]. The antitumor effect of PEITC has been discussed in numerous studies. In carcinogenesis, the epigenetic modification of DNA and histone proteins by methylation and deacetylation is one of the key factors [4]. A study by Cang et al. confirmed that PEITC can be used as a histone deacetylase (HDAC) inhibitor in various tumors (e.g., prostate cancer, breast cancer, leukemia, and myeloma cells) [5]. Hypoacetylated, hypomethylated, and dephosphorylated forms of the histone H2B in DU-145 prostate cancer cells can be reversed by HDAC inhibitors [6]. The inhibition of HDACs and DNA methyltransferases has been used as novel cancer therapy strategies for epigenetic modification in acute myeloid leukemia [7]. Although PEITC has shown promising anti-cancer effects in clinical trials on leukemia [8], the toxicity effects of PEITC were mainly evaluated on liquid tumors and not solid tumors. Our previous studies revealed the in vitro effects of PEITC on human glioblastoma GBM 8401 cells: (1) inducing apoptosis through the extrinsic (death receptor) pathway, dysfunction of mitochondria, reactive oxygen species (ROS)-induced endoplasmic reticulum (ER) stress, and the intrinsic (mitochondrial) pathway in GBM 8401 cells [9]; (2) suppressing migration and invasion through the inhibition of uPA, Rho A, and Ras with inhibition of MMP-2, -7, and -9 gene expression [10]; (3) altering the gene expressions and the levels of protein associated with cell cycle regulation [11]. However, the function of PEITC in various cancer-promoting mechanisms, including cell proliferation, progression, and metastasis, in living subjects with glioblastoma remains ambiguous.

There are no reports in the available literature disclosing that PEITC inhibits the growth of glioblastoma in vivo. In the present study, we first investigated the anti-tumor effects of PEITC in a xenograft model of glioblastoma in nude mice.

## 2. Results

### 2.1. PEITC Did Not Affect the Body Weights in Xenograft GBM 8401/luc2 Cells-Bearing Animal Models

**Figure 1:**
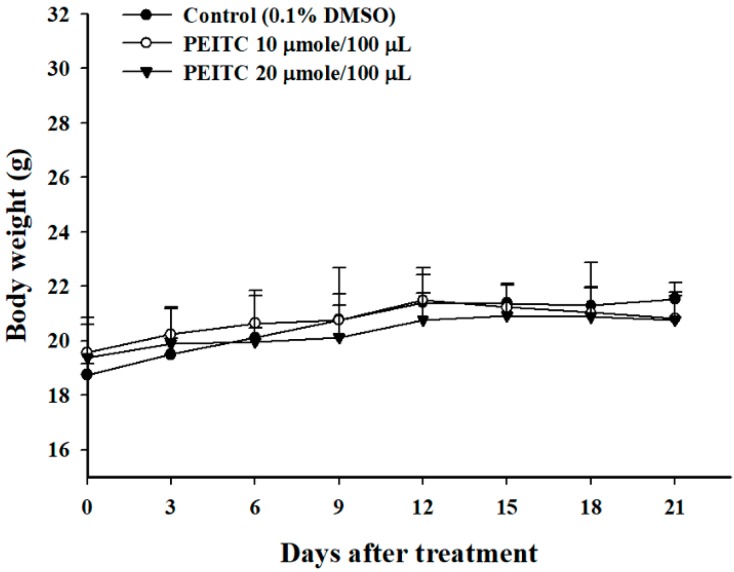
The effects on body weight in xenograft GBM 8401/*luc2* cells-bearing mouse models. The body weights of mice treated with phenethyl isothiocyanate (PEITC) remained similar to those of control mice throughout the study period.

The body weights of each group were measured every 3 days, and the results are shown in Figure 1. Notably, no significant difference of body weight change was seen among the control, PEITC-10 (10 μmole PEITC/100 μL PBS), and PEITC-20 (20 μmole PEITC/100 μL PBS) groups (Figure 1), which indicated no signs of acute or delayed toxicity of PEITC.

### 2.2. PEITC Inhibited Xenograft Tumor Growth of GBM 8401/luc2 Cells

**Figure 2:**
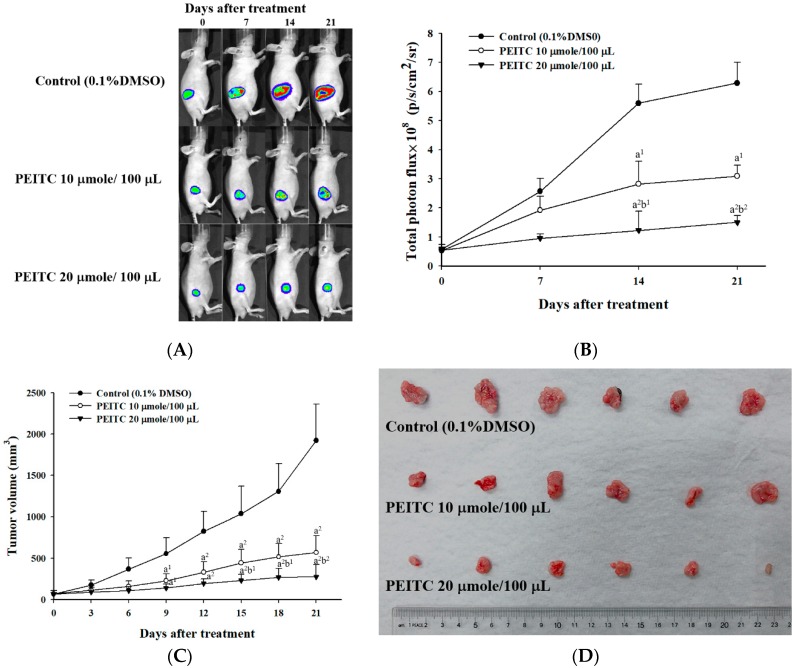

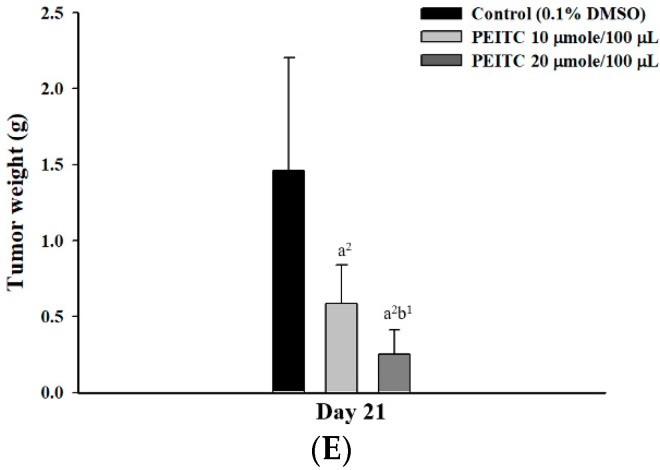
Therapeutic efficacy evaluation of PEITC in xenograft GBM 8401/*luc2* cells-bearing mice. (**A**) The tumor growth of each mouse was monitored by bioluminescent imaging (BLI) every one week. The tumor growth was significantly suppressed by PEITC at both doses (PEITC-10, PEITC-20) compared to the control group. (**B**) The regions-of-interest (ROIs) of tumors in (**A**) were quantified. The PEITC-20 group revealed the most obvious tumor inhibition. a^1^: *p* < 0.05, a^2^: *p* < 0.01 compared to that of the control; b^1^: *p* < 0.05, b^2^: *p* < 0.01 compared to that of PEITC-10 group. (**C**) The tumor volumes of each mouse were assayed by caliper measurement every 3 days. The tumor volumes were significantly reduced by PEITC at both doses (PEITC-10, PEITC-20 groups) compared to the control group. a^1^: *p* < 0.05, a^2^: *p* < 0.01 compared to that of the control; b^1^: *p* < 0.05, b^2^: *p* < 0.01 compared to that of PEITC-10 group. (**D**) Six representative tumor pictures from each group are displayed after the mice were sacrificed. (**E**) The tumor weights of each mouse were assayed after they were sacrificed on day 21. The tumor weights were significantly decreased by PEITC at both doses (PEITC-10, PEITC-20 groups) compared to the control group. a^2^: *p* < 0.01 compared to that of the control; b^1^: *p* < 0.05 compared to that of PEITC-10 group.

Ectopic tumor-bearing nude mice were treated with vehicle and PEITC at different concentrations for 21 days, and they were anesthetized with 1–3% isoflurane every 1 week during scanning. The efficacy of the treatment was evaluated by bioluminescent imaging (BLI) (Figure 2A). Photons emitted from the tumors of the PEITC-10 group were significantly lower than those of the control group, and those emitted from the tumors of PEITC-20 group were significantly lower than those of the PEITC-10 group (Figure 2B). These results suggested that both doses of PEITC reduced the total photon flux significantly in comparison with control group, and the higher dose of PEITC led to a lower total photon flux than did the lower dose of PEITC.

The tumor volume of each mouse was measured every 3 days during treatments for 21 days, and six representative tumors from three groups were extracted as shown in Figure 2C,D. These indicated that both doses of PEITC significantly decreased the tumor volumes in comparison with the control group, and the higher dose of PEITC resulted in lower tumor volumes than did the lower dose of PEITC. Both doses of PEITC also significantly reduced the tumor weights in comparison with the control group, and the higher dose of PEITC led to lower the tumor weights than did the lower dose of PEITC (Figure 2E).

### 2.3. PEITC Altered Apoptosis Associated Proteins Signaling in Xenograft Tumor of GBM 8401/luc2 Cells

**Figure 3:**
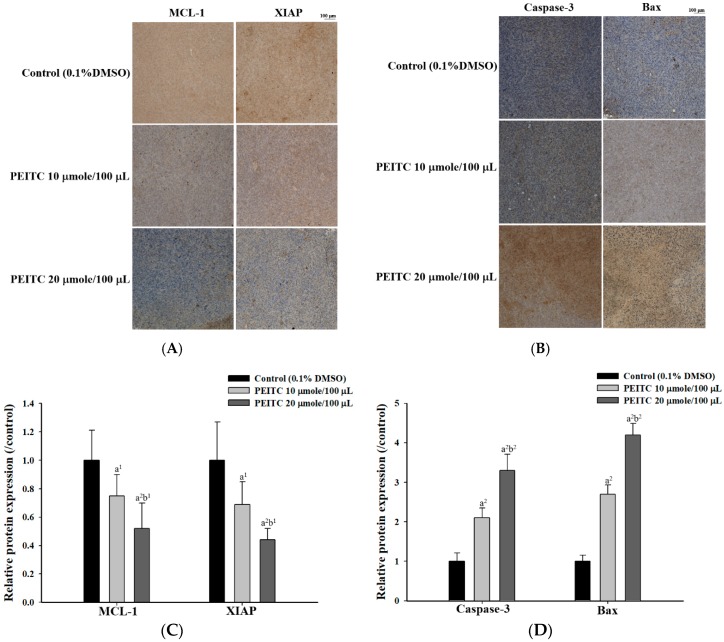
The effects of PEITC on the expressions of apoptosis-associated proteins in xenograft GBM 8401/*luc2* cells-bearing mice. Tumors were isolated from xenograft GBM 8401/*luc2* cells-bearing mice after treatment. All samples were analyzed under microscopy at ×100 magnification and photographed. (**A**) MCL-1 (myeloid cell leukemia 1) and XIAP (X-linked inhibitor of apoptosis protein); (**B**) Caspase-3 and Bax. (**C**) Quantification results of MCL-1 and XIAP. (**D**) Quantification results of Caspase-3 and Bax. a^1^: *p* < 0.05, a^2^: *p* < 0.01 compared to that of the control; b^1^: *p* < 0.05, b^2^: *p* < 0.01 compared to that of PEITC-10 group.

All samples were observed under microscopy at ×100 magnification after immunohistochemical (IHC) staining. Five regions of each slide were randomly selected for photographing (Figure 3A). Results indicated that the samples at both doses of PEITC were weakly stained with anti-MCL-1 (myeloid cell leukemia 1) and -XIAP (XIAP (X-linked inhibitor of apoptosis protein) compared to the control group (Figure 3A). The higher dose of PEITC (20 μmole/100 μL PBS/day) led to lower staining with anti-MCL-1 and -XIAP than the low dose of PEITC (10 μmole/100 μL PBS/day). The samples at both doses of PEITC were strongly stained with anti-caspase-3 and -Bax compared to the control group (Figure 3B). The higher dose of PEITC resulted in higher staining with anti- caspase-3 and -Bax than did the low dose of PEITC. The quantification of MCL-1, XIAP, caspase-3, and Bax proteins expression was performed by Image J software (Madison, WI, USA), respectively (Figure 3C,D). Thus, PEITC changed the expressions of apoptosis-associated proteins in the signal pathway of GBM 8401/*luc2* cells in vivo.

### 2.4. Effects of PEITC on the Hepatic Histopathological Change in GBM 8401/luc2 Cell Xenograft Animal Model

**Figure 4:**
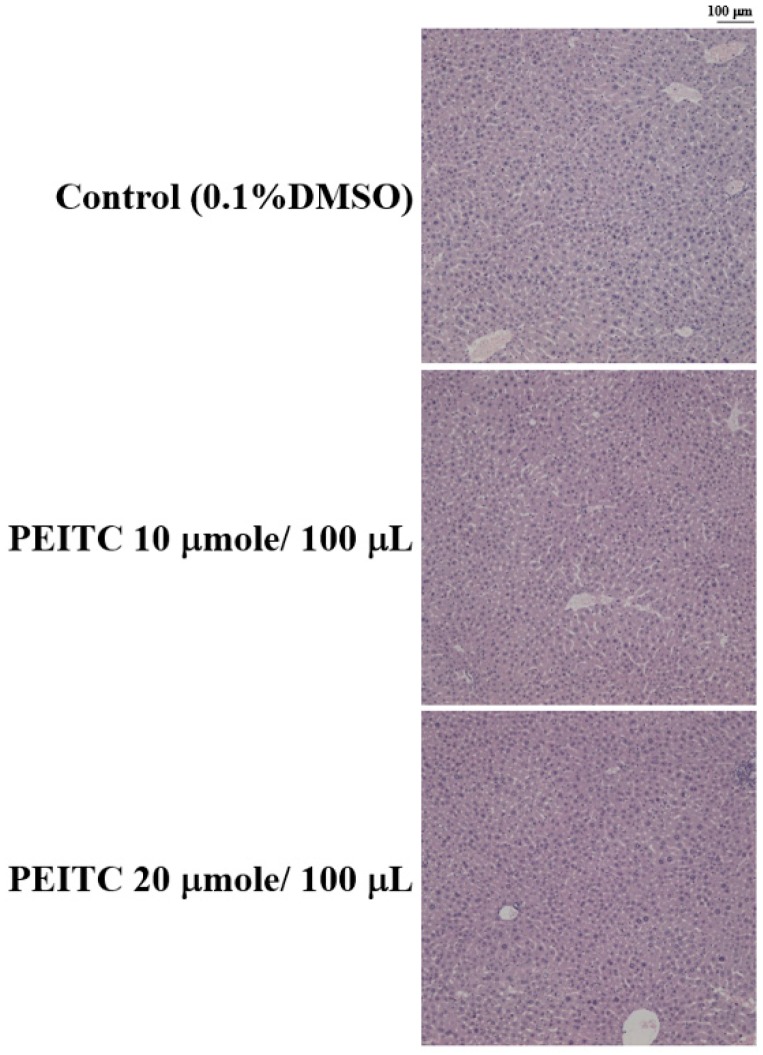
The effects of PEITC on liver histopathology in GBM 8401/*luc2* cells xenograft animal model. Liver tissue from every mouse of every group after treatment was collected and stained with hematoxylin and eosin (H&E). Liver specimens from PEITC-treated and control groups revealed similar structures of hepatocytes and lobular architectures.

Liver was collected from every mouse of every group after treatment, embedded in paraffin, sectioned into 5 μm-thick slices, deparaffinized, and stained with hematoxylin and eosin (H&E). Liver specimens from PEITC-treated and control groups revealed similar hepatocyte arrangement in hepatocytes and lobular architectures (Figure 4).

There were no significant differences in mice liver between PEITC-treated and control groups, so there was no obvious hepatic cytotoxicity after PEITC treatment GBM 8401/*luc2* cells in vivo.

## 3. Discussion

PEITC reduced tumor weights, but did not affect total body weights in subcutaneous xenograft tumors of human malignant melanoma A375.S2 cells-bearing mice in vivo [12]. In the present study, PEITC also did not affect total body weights in xenograft GBM 8401/*luc2* cells-bearing mice (Figure 1). However, PEITC did reduce the tumor growth in xenograft GBM 8401/*luc2* cells-bearing mice (Figure 2). PEITC had in vivo effects on different mouse cancer models [13]. The growth arrest of prostate cancer cells can be induced by miR-130b~301b cluster overexpression through the epigenetic regulation of proliferation-blocking genes and the activation of cellular senescence [14]. It was chemopreventive to normalize microRNAs of which downregulation was epigenetically induced by environmental cigarette smoke in a Sprague-Dawley rat lung cancer model [15]. In the azoxymethane (AOM)-initiated and dextran sodium sulfate (DSS)-promoted-C57BL/6 mice colon cancer model, PEITC inhibited colon tumor multiplicity and intestinal polyp development, and reduced intestinal tumor size associated with apoptosis (enhanced cleaved caspase-3 and-7) and cell cycle arrest (elevated p21) [16]. Therefore, PEITC had in vivo effects on different cancer models including glioblastoma, melanoma, lung, and colon cancers.

In our previous study, PEITC could induce apoptosis of human brain glioblastoma GBM 8401 cells through the extrinsic and intrinsic signaling pathways in vitro [9]. PEITC decreased anti-apoptotic protein MCL-1, and inhibited XIAP in vitro. In the present study, both doses of PEITC reduced the levels of MCL-1 and XIAP in GBM 8401 cells in vivo (Figure 3A). The anti-apoptotic MCL-1 is a key regulator in cancer cell survival, and can be a therapeutic target [17,18]. In the highly aggressive U87-EGFRvIII model and a patient-derived xenograft system, sorafenib suppressed MCL-1 expression by combining with validated compounds of histone deacetylase (HDAC) inhibitor and Bromodomain protein (BRD) inhibitor, and facilitated apoptosis from the combination treatment in vivo [19]. Additionally, PEITC is also known to act as a HDAC inhibitor in prostate cancer, leukemia, and myeloma cells [5,6,7]. HDAC inhibitors have been approved to treat T-cell lymphomas, and in many clinical trials for other hematologic and solid cancers (over 500 studies in clinicaltrials.gov) [20]. Multiple histone modifications changing global gene expression might be involved in certain cancers, so the effects of combination therapy targeting two or more associated epigenetic changes could be synergistic [20]. In murine model systems of patient-derived orthotopic xenografts of human glioblastoma, breast cancer, and melanoma in vivo, and human glioblastoma U87MG, LN229, U251, T98G cells in vitro, the tumor growth can be degraded by the inhibition of histone deacetylase, mitochondrial matrix chaperones, and anti-apoptotic B-cell lymphoma 2 (Bcl-2) proteins including Bcl-2, Bcl-xL, and MCL-1 [19,21,22]. PEITC also inhibited the tumor growth by inhibiting MCL-1 in our GBM 8401 ectopic xenografts in vivo (Figure 3A).

Inhibitor of apoptosis proteins (IAPs) are anti-apoptotic proteins including cIAP1 (cellular inhibitor of apoptosis protein-1), cIAP2 (cellular inhibitor of apoptosis protein-2), XIAP and ML-IAP (melanoma inhibitor of apoptosis protein), and facilitate treatment resistance by inhibiting caspase activation [23]. XIAP degradation was induced and the NF-κB pathway was inhibited by 3-((decahydronaphthalen-6-yl)methyl)-2,5-dihydroxycyclohexa-2,5-diene-1,4-dione (RF-Id), which led to the cleavage of caspases 8, 9, 3, and 7, and blocked c-IAP2/XIAP interaction in in human glioblastoma U87MG and LN229 cells in vitro [24]. The caspase-dependent apoptosis in glioblastoma cells can be induced by RF-Id by inhibiting IAP family proteins and the NF-κB pathway. GDC-0152, a SMAC (second mitochondria-derived activator of caspases) mimetic antagonizing these IAPs, affected human glioblastoma U87MG orthotopic xenografts in a dose-dependent manner. It delayed tumor formation, slowed down tumor growth in vivo, and thereafter improved the survival of GBM-bearing mice [25]. PEITC also inhibited tumor growth by inhibiting XIAP in our GBM 8401 ectopic xenografts in vivo (Figure 3A).

PEITC increased the levels of caspase-3, -9, -8, -2 and -4 of GBM 8401 cells in vitro [9], and both doses of PEITC elevated the levels of caspase-3 and Bax of GBM 8401 cells in vivo in the present study (Figure 3B). The higher dose of PEITC resulted in higher levels of caspase-3 and Bax in GBM 8401 cells in vivo. The induction of apoptosis involves the Fas receptor and the activation of initiator caspases (caspase-8 and -9) as well as an executioner caspase (caspase-3) [22,26,27]. The expressions of cell cycle regulator Cdk1 (cyclin-dependent kinase 1) and anti-apoptotic protein Bcl-2 were decreased, and the expression of Bax and cleavage of PARP (poly ADP ribose polymerase) proteins were increased by the synergistic effects of the epigenetic agent PEITC and the chemotherapeutic agent paclitaxel (taxol) in breast cancer cells [5]. The levels of pro-apoptotic Bax and cleaved caspase-3 were enhanced, and Bcl-2 expression was downregulated from leucine-rich α2 glycoprotein 1 (LRG1)-silencing, which inhibited the growth of xenograft tumors and induced apoptosis of U251 glioblastoma cells in vitro and in vivo [28]. The increased levels of caspase-3 or Bax can be discovered in the apoptosis of different glioblastoma cell lines in vitro and in vivo. PEITC inhibited tumor growth by enhancing caspase-3 and Bax in our GBM 8401 ectopic xenografts in vivo.

In the present study, there were no significant differences in mouse liver between PEITC-treated and control groups, so there was no obvious hepatic cytotoxicity after PEITC treatment of GBM 8401/*luc2* cells in vivo (Figure 4). In the Sprague-Dawley rat model, the activity and protein levels of hepatic glutathione S-transferases (GSTs) were increased in a dose-dependent manner after treatment with PEITC [29]. On the contrary, PEITC may have a protective function against hepatotoxicity of acetaminophen through its induction effect on GST. It might be safe to use PEITC in animal model of glioblastoma and the development of potential anticancer agents.

Taken together, PEITC can diminish the ectopic xenograft tumor growth of GBM 8401 cells in tumor weights and volumes, which may be through the induction of apoptosis by the decrease of anti-apoptotic proteins MCL-1 and XIAP, and the increase of pro-apoptotic proteins caspase-3 and Bax. The in vivo effects of PEITC on the growth of GBM 8401 cells might contribute to new insights into anti-tumor treatment for glioblastoma.

## 4. Materials and Methods

### 4.1. Chemicals and Reagents

PEITC, Tris-HCl, trypan blue, and dimethyl sulfoxide (DMSO) were purchased from Sigma Chemical Co. (St. Louis, MO, USA). RPMI-1640, fetal bovine serum (FBS), L-glutamine, penicillin-streptomycin, and trypsin-EDTA were obtained from Gibco BRL/Invitrogen (Carlsbad, CA, USA). The primary antibodies and secondary antibody, anti-MCL-1 (myeloid cell leukemia 1), anti-XIAP (X-linked inhibitor of apoptosis protein), anti-caspase-3, and anti-Bax, and anti-goat IgG were obtained from Cell Signaling Technology (Irvine, CA, USA) and Amersham Pharmacia Biotech, Inc. (Piscataway, NJ, USA), respectively. PEITC was dissolved in DMSO.

### 4.2. Cell Culture

Human brain glioblastoma multiforme (GBM 8401) cell line was purchased from the Food Industry Research and Development Institute (Hsinchu, Taiwan) and cultured according to the provider’s instructions. Cells were cultured in RPMI 1640 medium supplemented with 10% fetal bovine serum (FBS), 2 mM L-glutamine, 100 Units/mL penicillin, and 100 μg/mL streptomycin and grown at 37 °C under a humidified 5% CO_2_ and 95% air at one atmosphere. The medium was changed every 2 days [30].

### 4.3. Transfection and Stable Clone Selection

We transfected GBM 8401 cells with the plasmid of pGL4.50 luciferase reporter (pGL4.50[*luc2*/CMV]) vector using JetPEI™ transfection reagent (Polyplus transfection, New York, NY, USA) [31]. The plasmid of pGL4.50 luciferase reporter (pGL4.50[*luc2*/CMV]) was obtained from Promega (Madison, WI, USA). Stable clone was selected by two-week treatment of 200 μg/mL hygromycin and validated by a Xenogen IVIS imaging system (Xenogen, Alameda, CA, USA). Luciferase expressing stable clone was finally named as GBM8401/*luc2*.

### 4.4. Animals and Treatments

**Figure 5:**
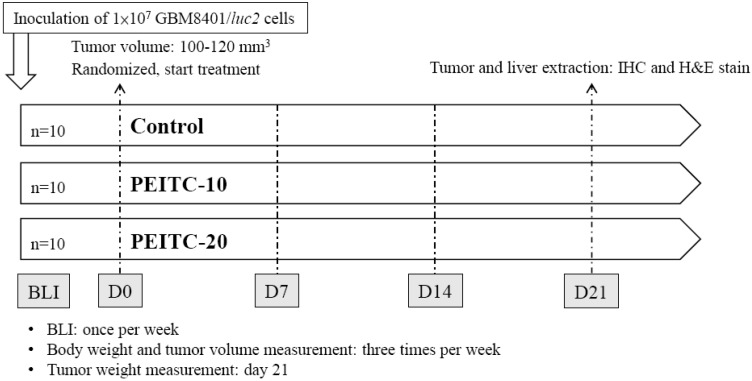
Experimental design for the treatments of human GBM 8401-bearing mice. Each mouse was injected with 1 × 10^7^ GBM 8401/*luc2* cells. After the tumor volume reached 100–120 mm^3^, mice were randomized into three different treatment groups (n = 10 per group). PEITC (10, 20 μmole/100 μL PBS) was administered daily by gavage. All mice were sacrificed 21 days after treatments. IHC: immunohistochemical.

Six-week-old male athymic CAnN.Cg-*Foxn1*^nu^/CrlNarl nude mice were bought from the National Laboratory Animal Center, Taipei, Taiwan. All studies followed the National Institutes of Health Guidelines for Animal Research, and were approved by the Institutional Animal Care and Use Committee of Taipei Medical University (number: LAC-2017-0248). GBM8401/*luc2* cells (1 × 10^7^) in 150 μL mixture containing serum-free RPMI and Matrigel (2:1) were subcutaneously inoculated into the right hind legs of the 30 mice [32]. We measured the tumor volume of each animal with a digital caliper and calculated with the equation: tumor volume = 0.523 × length × width^2^ [33]. After the tumor volume reached 100–120 mm^3^, mice were randomized into three different treatment groups (n = 10 for each group), including vehicle, PEITC-10 group, and PEITC-20. The vehicle group was treated with 110 μL phosphate-buffered saline (PBS) plus 10 μL DMSO by gavage daily for 21 days. PEITC-10 and PEITC-20 groups were treated with PEITC 10 μmole/100 μL PBS/day and PEITC 20 μmole/100 μL PBS/day for 21 days, respectively. We monitored the tumor growth with bioluminescent imaging (BLI) and caliper. The body weights and tumor volumes of mice were measured 3 times per week after treatment. Finally, livers and tumors extracted from mice were prepared for pathologic examination and immunohistochemical (IHC) staining on day 21. Tumor weights of mice were also recorded. The flow chart of our experimental protocol is summarized in Figure 5.

### 4.5. In Vivo Bioluminescent Imaging (BLI)

Mice tumor growth was also monitored by BLI once per week during treatment progress. Intraperitoneal injections of 150 mg/kg of D-luciferin (Promega, Madison, WI, USA) were administered to mice from each group 15 min before BLI scanning. During the scanning procedure, mice were anesthetized at 1–3% isoflurane dosage and emitted photons were recorded by a Xenogen IVIS imaging system 200 as described previously [32]. The acquisition period was 1 s, and then the signals emitted from the regions of interest were quantified by Living Image software (Version 2.20, Xenogen, Alameda, CA, USA) [34].

### 4.6. IHC Staining and Pathological Examination

Mice were sacrificed on day 21, and tumors and livers were extracted. Tumors and livers were both fixed with 4% paraformaldehyde (PFA) at 4 °C for 24 h. Paraffin-embedded tumor tissues and liver tissue were sliced at 5 μm thickness by Bio-Check Laboratories Ltd. (New Taipei City, Taiwan). The IHC and hematoxylin and eosin (H&E) staining protocols were performed according to the manufacturer’s recommendations. For immunohistochemical staining, primary antibodies including MCL-1, XIAP, Bax, and active Caspase-3 antibodies were added, respectively. An H&E staining kit from Bio-Check Laboratories Ltd. was used to stain slices. All slices were finally mounted with Prolong Gold Antifade reagent (ThermoFisher Scientific, Waltham, MA) and were imaged by microscopy-based TissueFAXS platform (TissueGnostics, Vienna, Austria) at 100× magnification [35]. Positive expression of MCL-1, XIAP, caspase-3, and Bax on IHC indices in tumor tissues was quantified with ImageJ software version 1.50 (National Institutes of Health, Bethesda, MD, USA) [36].

### 4.7. Statistical Analysis

All data were represented with the mean ± standard error. One-way ANOVA with Newman–Keuls multi-comparison test was used for the comparison between PEITC-treated and control groups, and between PEITC-treated groups. Difference between the means was considered significant if *p* < 0.05 or less.
